# Examining the environmental consequences of China's mineral wealth and governance in the context of the natural resource curse

**DOI:** 10.1016/j.heliyon.2024.e35891

**Published:** 2024-08-10

**Authors:** Heshu Qiu

**Affiliations:** a Xinyang Normal University, Business School, Xinyang, 464000, China; b Reserch Institute of the Economic and Social Development in the dabie Mountains, Xinyang, 464000, China

**Keywords:** Natural resources, Governance, Environmental consequences, Sustainable, Financial

## Abstract

This study investigates the environmental consequences of China's mineral wealth and governance within the framework of the natural resource curse. The natural resource curse posits that countries rich in natural resources often experience less economic growth and worse development outcomes than countries with fewer natural resources. This phenomenon is particularly relevant for China, which has experienced significant mineral wealth since the 1990s. The primary aim of this research is to assess how China's mineral wealth and governance structures have influenced environmental outcomes from 1990 to 2022, using an econometric model. The results reveal three key findings: First, regions with higher mineral wealth have experienced more severe environmental degradation, particularly in terms of air and water pollution. Second, governance quality plays a critical role in moderating these effects, with better governance associated with less environmental harm. Third, economic growth driven by mineral wealth does not necessarily translate into improved environmental conditions, highlighting a disconnect between economic and environmental objectives. The policy implication is clear: to mitigate the environmental consequences of mineral wealth, China must strengthen its governance mechanisms and integrate environmental considerations into its resource management strategies.

## 1 Introduction

The problem this study addresses is the significant environmental degradation observed in China, which is often attributed to its vast mineral wealth and the accompanying challenges of governance. Despite the country's rapid economic growth fueled by abundant natural resources, there is a concerning trend of deteriorating air and water quality, loss of biodiversity, and increasing pollution levels. This phenomenon aligns with the natural resource curse theory, which posits that resource-rich countries often suffer from poor economic and development outcomes due to factors such as corruption, weak institutions, and inadequate governance. In China's case, the extraction and processing of minerals have led to severe environmental impacts, raising questions about the role of governance in either exacerbating or mitigating these effects. The study seeks to dissect the intricate relationship between mineral wealth, governance quality, and environmental outcomes to understand why economic prosperity driven by natural resources often fails to translate into sustainable environmental practices. By examining the specific governance structures and policies in place, this research aims to identify the underlying causes of environmental degradation in mineral-rich regions and propose effective strategies to harmonize economic development with environmental sustainability Ullah et al., 2024 [1]

This study's main goal is to provide a comprehensive analysis of the environmental effects linked to China's significant mineral riches and the governance frameworks controlling its extraction. By doing this, the research aims to determine if China is vulnerable to the "Natural Resource Curse," a phenomenon in which an abundance of natural resources has a negative influence on the environment and impedes the advancement of sustainable development Chen et al., 2024[2] The goal of the study is to provide a comprehensive knowledge of the intricate relationships that exist between China's mineral richness, the legal frameworks that govern its exploitation, and any possible negative effects on the environment. The study aims to provide comprehensive insights that can educate policymakers, environmental advocates, and academics about the opportunities and challenges of managing mineral resources sustainably in the context of China's distinct socio-economic and ecological landscape [3] [4]. This is achieved by closely examining the current governance mechanisms and environmental outcomes. Focusing on China's governance procedures and abundant mineral resources, this research explores the country's twin dynamics of economic expansion and environmental effects. Historically a major force behind economic growth, the banking industry has aided in environmental deterioration by funding initiatives that threaten ecosystems. In response, the Chinese government has put policies into place. One such program is the green finance program, launched by the People's Bank of China (PBOC) in 2016 to encourage lending practices and environmentally beneficial projects. Based on empirical data, China's green economic development has been favorably impacted by these initiatives, highlighting the critical role that sustainable finance plays [5].

Additionally, the research explores the important topic of natural resource management. Ecosystems have suffered due to China's exploitation of its natural resources, even though they have historically been crucial for economic growth [[6], [7]]. In response, the government has enacted policies to improve resource efficiency, including encouraging renewable energy sources and environmentally friendly mining techniques. China's quest for ecological civilization and sustainable development requires an understanding of the complex link between economic growth and natural resource efficiency (M. [8]). In addition to adding to the body of knowledge by conducting an in-depth empirical investigation of this link using rigorous econometric analysis, the study has applications for environmentalists, corporate executives, and legislators. It aims to close the knowledge gap on how China's economic growth is directly impacted by sustainable resource management. The research seeks to facilitate the creation and implementation of effective policies that maintain a harmonic balance between environmental preservation and economic advancement by offering evidence-based insights (H. [9]). This study has important worldwide ramifications as China's economic development shapes the global ecological scene. Through analyzing the intricacies of resource allocation and economic development in one of the global economy's biggest economies, this research seeks to provide insightful guidance for other countries with comparable difficulties. To create a solid framework for comprehending the connection between resource efficiency and economic development in China, the methodology places a high priority on objectivity and dependability.

This study makes several significant contributions to the understanding of the environmental consequences of mineral wealth in the context of the natural resource curse, focusing on China from 1990 to 2022. Utilizing an econometric model, the research provides a comprehensive analysis of how mineral wealth and governance quality influence environmental outcomes. First, it extends the literature on the natural resource curse by specifically examining its environmental dimension in a rapidly developing country. Second, the study employs a robust econometric approach, allowing for precise estimation of the relationships between mineral wealth, governance, and environmental indicators such as air and water pollution. Third, by covering a substantial time period of over three decades, the research captures the dynamic changes in China's economic and environmental landscape, offering insights into long-term trends and patterns. Fourth, the regional focus on China, a major global player with significant mineral resources, provides valuable case-specific insights that can inform both national and international policy discussions. Lastly, the study's findings emphasize the critical role of governance in mitigating negative environmental impacts, suggesting that improvements in governance quality can significantly alleviate the adverse environmental effects of mineral wealth. These contributions collectively enhance our understanding of the complex interplay between natural resources, governance, and environmental sustainability.

## 2 Literature review

It is becoming increasingly clear that nations must set out on a sustainable recovery path in the wake of global problems. To promote a green economic recovery, this study explores the importance of visualizing and aligning with the Sustainable Development Goals (SDGs). In addition to highlighting the interdependence of social equity, resource efficiency, and environmental preservation, the research examines evidence-based approaches to nation-building that are more resilient and sustainable (W. [10]). The research endeavors to provide innovative perspectives on navigating the post-crisis terrain via the utilization of scholarly papers and reports. The research focuses on the complex link that exists between a country's natural resource-based riches and its effect on sustainable development. Many studies have been conducted to examine the impact of Natural Extraction Revenue (NRR) on environmental health Mohsin Muhammad, Dilanchiev Azer, 2023 [11] Notably, research shows that while China's early economic expansion raised demand for natural resources, it also increased costs associated with biological variety and a bigger ecological imprint. The research also looks at how, despite their economic advantages, the widespread use of natural gas and petroleum in certain areas has distorted the ecosystem. On the other hand, it has been shown that sustainably managed natural resources have a favorable effect on environmental sustainability. The paper delves further into how the banking industry has influenced China's green economy. Concentrating on the transformation of China's banking sector from a state-run to a more market-driven framework in the 1980s, the study highlights the critical role that big state-owned banks like ICBC, ABC, BOC, and CCB played in the nation's economic growth. Increased financial accessibility has been particularly advantageous for small and medium-sized enterprises (SMEs), promoting inclusivity [12]. 10.13039/100014337Furthermore, the People's Bank of China (PBOC)'s enforcement of regulations and the government's support of environmentally friendly businesses emphasize the industry's role in sustainable growth.

The study recognizes that natural resources, which comprise over 10 % of China's GDP, are essential for fostering economic growth. However, it also addresses the environmental issues resulting from their overuse, emphasizing the need for sustainable practices (Q. [13]). This commitment is evident in the government's initiatives to reduce the damaging consequences of natural resource extraction on the environment, such as emission limitations, pollution penalties, and environmental impact assessments. By analyzing the intricate interactions between social inclusion, ecological preservation, and economic growth, the research contributes to the discourse on green economic development. The study aims to guide governments, companies, and environmentalists in preventing environmental degradation while maximizing natural resource use for a more resilient and sustainable future.

### 2.1 Carbon dioxide emissions and energy consumption

The intricate relationship between energy usage and carbon dioxide emissions has been the subject of a protracted discussion in environmental and energy research. A key strategy for enhancing ecological sustainability in the face of climate change is using renewable energy sources, which lower CO2 emissions into the atmosphere [14]. Around the world, decision-makers have highlighted how using renewable energy can meet energy demands and lessen the harmful consequences of carbon dioxide emissions. By analyzing the connection between carbon dioxide emissions and the utilization of renewable energy in the BRICS nations (M. [15]), built on this issue. Their research, which made use of Vector Error Correction Models (VECM), showed that there was a reciprocal link between CO2 emissions and the usage of non-clean energy and the adoption of clean energy in OECD countries Ahmad et al., 2020 [16] This outcome aligns with the belief that incorporating more renewable energy sources into the energy mix often reduces overall carbon emissions. This notion was further reinforced by experimental tests conducted by Ref. [17] on the link between carbon dioxide emissions and the generation of green power.

Their research results provided empirical evidence for the negative relationship between carbon emissions and the use of renewable energy sources. The impact of transitioning to renewable energy on the nation's greenhouse gas emissions was also investigated, and the findings consistently indicated a negative correlation (C. [18]). Researchers extended their work to a European context and examined the association between CO2 emissions and the utilization of renewable energy in different countries using simultaneous spatial modeling. The findings supported the hypothesis that there is an inverse relationship between greenhouse gas emissions and the utilization of renewable energy sources. The geographic scope was widened by (F. [19]) examination of the effect of non-clean energy consumption on carbon dioxide emissions in West Africa. Their research demonstrated the harm that using unclean energy sources does to the environment, underscoring the need to switch to more sustainable and clean energy sources. These studies add to the increasing amount of data highlighting how important it is to use renewable energy sources to reduce carbon dioxide emissions and promote environmental health.

### 2.2 Development of the banking industry and emissions of carbon dioxide

Empirical research has focused on the interaction between economic market development, industrial production, and energy consumption, providing important insights into the complex relationships within these areas Umair & Dilanchiev, 2022 [20] examined how economic development affected GDP growth. They found a significant correlation between loans and spending from the private sector and greater energy consumption and carbon dioxide emissions [21]. This emphasizes how important financial activity is in determining patterns of energy usage. In a more comprehensive analysis (Y. [22]), confirmed that economic activities have a major influence on environmental outcomes by examining the financial sector's large effect on CO2 emissions (Y. [18]). discovered a significant relationship between economic growth and CO2 emissions by concentrating on China's energy consumption, economic development, and greenhouse gas emissions (M. [23]). Their findings highlighted the significant influence that Turkey's expanding banking sector has had on the nation's rising energy requirements, highlighting the relationship between the expansion of the financial industry and energy use patterns. This research advances our knowledge of how financial and economic factors may dramatically alter energy consumption under various conditions. These empirical investigations demonstrate the complex relationships that exist between the growth of economic markets, industrial production, and energy use. The results highlight the need to take financial activities and economic development trajectories into account when evaluating environmental impacts, highlighting the necessity of energy-efficient and sustainable practices in the face of growing economies.

Furthermore, the banking industry's contribution to CO2 emissions may vary depending on the specifics of the financial sector and the regulatory environment. Of the 38 nations that comprise the International Organization for Economic Cooperation and Development, some are among the most developed in the world. These diverse conditions must be considered while analyzing the link between economic development, the growth of the banking industry, and CO2 emissions in different geographic locations.

## 3 Data and methodology

The study conducted by Refs. Xiuzhen et al., 2022 [24] examined the connections among technological innovation (TI), natural resources, globalization, GDP expansion, economic development, and China's ecological footprint from 1990 to 2022. They achieved this by using annual intensity translational data. The authors used a comprehensive approach incorporating the rents from forests, oil, coal, natural gas, and petroleum to compute the total Natural Extraction Revenue (NRR), expressed as a percentage of GDP growth. The environmental effect was measured regarding Renewable Energy Certificates (RECs), while new patent filings functioned as a gauge for technical progress. With the World Development Indicators, NR calculations and the assessment of a country's financial infrastructure by GDP per capita were performed. The Urbanization (URB) and Environmental Footprint (EF) values were given by the KOF Internationalization Index, and the REC data was obtained from the Global Footprint. The World Intellectual Property Organization published the information about TI [25]. Kurtosis values, which display a combination of positive and negative skewness, were found to be less than three in both national and provincial panels, suggesting platykurtic variables. The Jarque-Bera statistic was used to demonstrate that the dataset was nonnormality-free. Notably, there was no evidence of Multicollinearity; variance inflation factors for the national and provincial panels were less than 5, as reported by Refs. Manaugh & El-Geneidy, 2011 [26] Descriptive data from the series illustrate developing nations' environmental footprints between 2001 and 2017. The study highlights a striking correlation between financial development and its ecological impact, highlighting the essential link between both factors.

The variables utilized in the research are explained in (Table 1), where each variable is represented by a symbol and is accompanied by the name of the associated variable and the primary data source. Log REO (Renewable Energy Out), the first variable, is generated from data found in the Chinese Statistical Almanac. It gathers data on China's production and use of renewable energy sources. The Yearbooks of Provincial Statistics provide the basis for the second variable, Log GNI (Gross National Income), which measures the nation's overall economic output. The Chinese Statistics Yearbook is the source of statistics for Environmental Resource Rents (ERR), which are expressed as Log ERR and pertain to the economic returns that arise from environmental resources. The fundamental data for Technology Adoption Rates (TAR) is taken from Provincial Statistics Yearbooks and is represented as log TAR (H. [27]). This variable shows how modern technologies are adopted and incorporated into different economic sectors.

**Table 1:** A description of the variables.

Symbol	Var.	Foundation
Log REO	Renewable energy out	The Chinese Statistical Almanac
Log GNI	Gross National Income	Yearbooks of Provincial Statistics
Log ERR	Environmental Resource Rents	Chinese Statistics Yearbook
log TAR	Technology Adoption Rates	Provincial Statistics Yearbooks
Log UPD	Urban Population Density	China's Environmental Statistics Yearbook

Finally, Log UPD represents Urban Population Density using information from China's Environmental Statistics Yearbook. This variable is essential for comprehending the demographic landscape and its possible effects on environmental issues since it sheds light on the population's concentration in metropolitan regions. When combined, these factors provide a thorough set of metrics for evaluating the complex interactions that exist in the Chinese setting between the use of renewable energy, economic production, rents from natural resources, adoption of new technologies, and urban population density. A further explanation for Turkey's increasing energy needs would include a combination of factors such as rapid industrialization, population growth, and urbanization. Even if the growing banking industry is recognized as a significant factor, there may be more variables influencing the country's rising energy use [28]. The outcomes of empirical studies on the relationship between CO2 emissions and economic growth might be influenced by various factors. Geographical variations, energy composition, and governmental laws, for instance, may all have a big influence on how different economies form their carbon footprints.

**Fig. 1:**
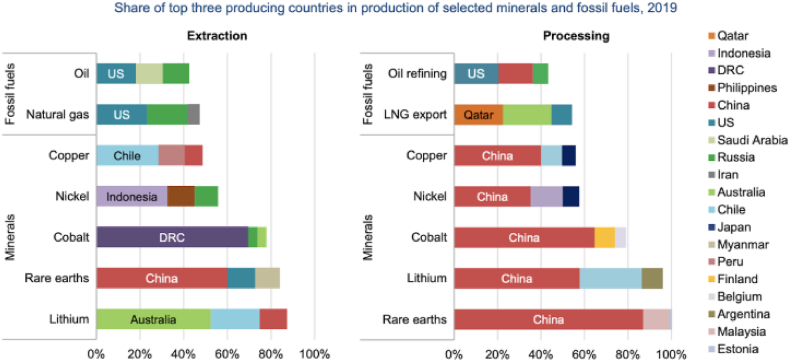
Mineral resources.

China leads in mineral production, the US is ahead of natural gas, and Saudi Arabia follows oil. This concentration of production in a few countries lends itself to vulnerabilities along the supply chain. It highlights China's increasing clout over vital materials such as rare earth metals, thereby supporting the need for diversification solutions and approaches as shown in Fig. 1.

However, it seems that there is minimal connection between human prosperity and globalization. It is important to remember that in this specific case, the rents from forests, oil, coal, natural gas, and petroleum are added to estimate the overall revenue from natural extraction. This is similar to what is frequently called the "natural resource curse" in literature, which emphasizes the potential disadvantages of relying too much on natural resources to fuel economic expansion.

### 3.1 Theoretical background

Information from several sources was combined to create a complete dataset covering 1992 to 2022. In this research, the average annual percentage increase over time is represented by the symbol Gi and is given as a logarithm when GDP per capita hits zero. In this collection, Li and Wang (2023) [29] address some subjects, such as spending, education, and dishonesty. A measure for determining the quantity or scarcity of natural resources is the vector Ri. An analysis of the primary sector data is shown in Table 1, which indicates a positive relationship between income growth and endowments with natural resources (Y. [30]). This emphasizes how crucial it is to consider various variables and industries when evaluating the dynamics of economic development, with a focus on the tertiary sector's significant contribution in this specific setting in equation (1).

(1 $$G_{i}=\alpha +\beta Y_{i,0}+\gamma R_{i,0}+\varphi Z_{i}+\varepsilon_{i}$$

This finding indicates a change in the impact of availability development over time since it stands in sharp contrast to the detrimental effects found between 1993 and 2019. It's interesting to note that this result adds credence to the idea that fossil fuel reserves have a favorable impact on economic dynamics Kongbuamai et al., 2020 [31] The three sectors' average annual absolute price indices were used to demonstrate a significant beneficial effect of natural resource availability on capital inflows over the previous global average pricing indices. Within the framework of this investigation, the noted helpful influence of the availability of natural resources on economic results is consistent with the "Natural Resource Curse." This phrase captures the phenomena where a region's richness of natural resources may cause more problems than advantages for the economy, such as heightened reliance, unstable economic conditions, and impediments to the growth of other industries. In contrast to the conventional story of the "curse of the natural resource" (NRC), the unanticipated positive effect, in this case, emphasizes the complex and dynamic link between resource availability and economic results.

**Fig. 2:**
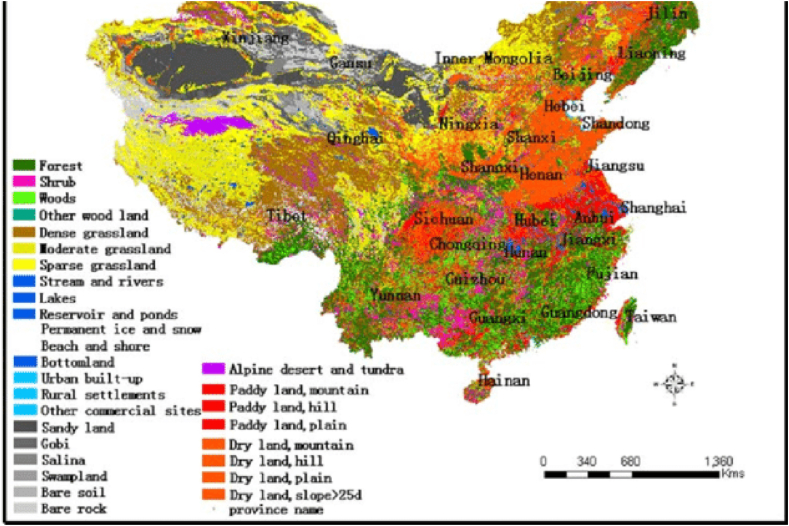
Spital distribution.

China is dominated by forest land, while mountains, grasslands, and deserts give way to regional diversity. This distribution had essential effects in terms of climate, habitat and availability of resources, but human activities such as deforestation and desertification have affected this pattern. China's policies focus on preserving the land currently used and encouraging sustainable practices for a productive future as shown in Fig. 2.

### 3.2 Model structure

The goal of the study was to comprehend how these factors interact and affect the Chinese economy. The inquiry focused on how China's economic trajectory is shaped by various factors, including technological innovation, globalization trends, the accumulation of human capital, and the growth of the financial sector. Natural Extraction Revenues are a form of income derived from natural resources. The research sought to provide insights into the complex dynamics impacting China's overall economic growth by evaluating these aspects in equation (2).

(2) $$R{\text{EO}}_{\text{it}}=A_{0}{\text{RER}}_{\text{it}}^{\beta_{1i}}{\text{TAR}}_{\text{it}}^{\beta_{2i}}{\text{UPD}}_{\text{it}}^{\beta_{3i}}{\text{GDPGNI}}_{\text{it}}^{\beta_{4i}}\mu_{\text{it}}$$

Equation (1) yielded a thorough calculation of the environmental cost footprint, and natural logarithms were used to represent each research component. Realistically examining the Environmental Kuznets Curve (EKC) theory was the main goal of the research. The link between economic growth and environmental damage could be thoroughly examined thanks to this modeling technique, which also tested the idea that environmental degradation may first rise and then reduce over time as a result of economic development [32]. The research sought to provide important insights into the intricate relationships between economic activity, environmental effects, and the suitability of the EKC hypothesis in light of China's developing economy via this analytical framework in equation (3).

(3) $$\ln\left({\text{REO}}_{\text{it}}\right)=\beta_{0\text{it}}+\beta_{1\text{it}}\;\ln\left({\text{RER}}_{\text{it}}\right)+\beta_{2\text{it}}\;\ln\left({\text{TAR}}_{\text{it}}\right)+\beta_{3\text{it}}\;\ln\left({\text{UPD}}_{\text{it}}\right)+\beta_{4\text{it}}\;\ln\left({\text{GNI}}_{\text{it}}\right)+\mu_{\text{it}}$$

This study's main goal was to determine if Natural Extraction Revenues and technological improvements help Chinese countries have less of an adverse environmental effect. Essentially, the goal of the study was to comprehend how advances in natural resource management and technology may help mitigate negative environmental impacts [33]. To guarantee the validity of the results, the research used many models for vulnerability assessment. Equation (3) had two changes, as described by Bourcet and Bovari (2020). First, a phrase was added to describe the exchange of information between information technology (IT) and natural resource management.

With this innovation, we want to capture better the synergistic and collaborative impacts that these two important components may have. Secondly, GDPSQ was not included; instead, a linear model description was used. This shift in the modeling approach focused on the direct effects of technical innovation and Natural Extraction Revenues on environmental outcomes to present a more understandable and straightforward depiction of the linkages under investigation in equation (4).

(4) $$\ln\left({\text{REC}}_{\text{it}}\right)=\beta_{0\text{it}}+\beta_{1\text{it}}\;\ln\left({\text{NRR}}_{\text{it}}\right)+\beta_{2\text{it}}\;\ln\left({\text{TI}}_{\text{it}}\right)+\beta_{3\text{it}}\;\ln\;{\left(NRR*TI\right)}_{it}+\beta_{4\text{it}}\;\ln\left({\text{URB}}_{\text{it}}\right)+\beta_{5\text{it}}\;\ln\left(G_{\text{DPit}}\right)+\mu_{\text{it}}$$

To provide a comprehensive comparison, we performed projections on subsamples of China for equations (3), (4). Examining the robustness and consistency of the results across provinces while taking into account the microeconomic differences within developing nations was the goal of this technique (H. [27]). For this reason, an analysis of microeconomic subsamples is crucial. Even though every country in the research is classified as "developing," there are notable differences in the rates of economic advancement. For example, there were significant disparities in GDP per capita in 2016. A more nuanced understanding of how technological innovation and Natural Extraction Revenues may interact with environmental consequences within the varied economic landscapes of Chinese provinces is therefore made possible by breaking down the data into subsamples.

**Fig. 3:**
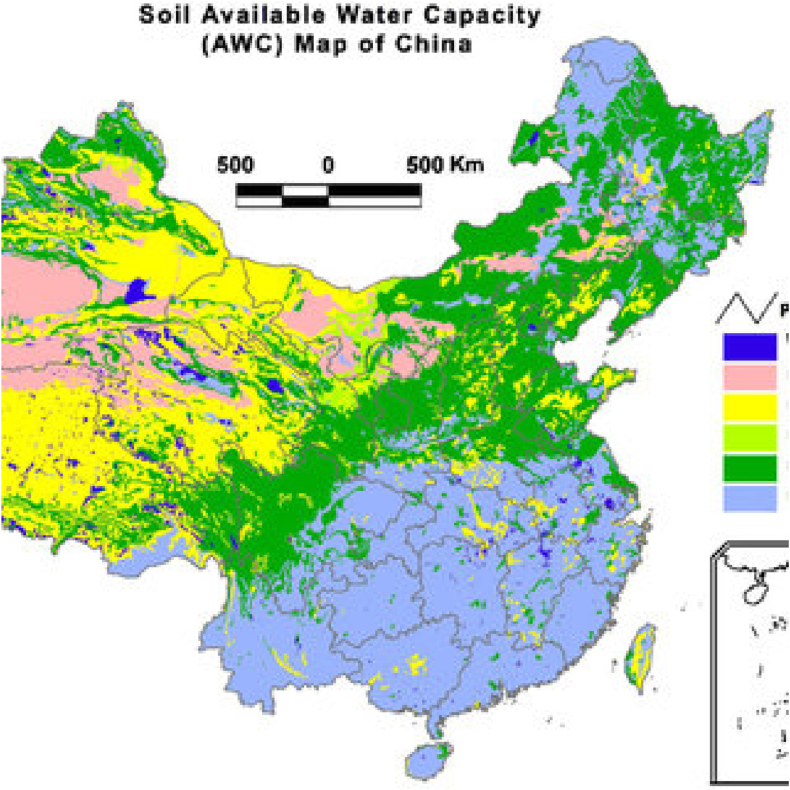
Water holding capacity.

China's soil water availability reveals a tale of two extremes: lush, well-watered south and east against drier northwest. Rainfall and soil are the major factors determining this variance, which significantly affects agriculture; high AWC areas thrive while drier regions need irrigation or face off droughts. Recognizing this, measures of water conservation and irrigation efficiency are taken by China to secure efficient food production in its varied landscape as shown in Fig. 3.

### 3.3 Methodology framework

Four separate phases comprised the systematic advancement of the study design. First, cross-sectional dependence (CSD) was analyzed in detail using the [34]technique as a guide. To provide a thorough grasp of the linkages at work, this stage examined any possible interdependencies and interactions between various dataset portions or units. Studying unit roots within the variables under consideration was part of the second step. The objective of this stage was to determine each variable's stationarity, which is essential for guaranteeing the accuracy and resilience of ensuing studies. Cointegration analysis was used in the third stage of the research to examine the long-term connections between the variables. This stage provided important insights into the dynamics of the phenomena under study by revealing any potential lasting linkages. Regression analysis, which uses statistical models to evaluate the correlations and dependencies between variables, was the fourth and last phase of the study. In this respect, it is noteworthy that natural resource abundance, a measure of the availability and exploitation of natural resources within the investigated regions, was one of the major factors evaluated in the research Omri & Bel Hadj, 2020 [35] This variable was crucial in explaining how different economic and environmental elements were affected by resource endowments. The study sought a thorough and rigorous investigation of the relationship between the quantity of natural resources, economic factors, and environmental effects across these four phases.

### 3.4 Evaluation of interconnectedness in cross-sectional data

Cross-sectional dependencies (CSDs) in long-term datasets might add bias and uncertainty. Therefore, rigorous assessment procedures are required to guarantee the studies' robustness. When certain traits or attributes are present in every cross-sectional unit, there are issues related to CSDs. Notably, the natural resource variable was crucial to this assessment methodology. It was essential to comprehend how the interdependencies affected the dataset since it clarified the effects of several variables, including location, industrialization, and financial cooperation, on the quantity and use of natural resources in the areas under study. By addressing possible CSD concerns, the research sought to assure the validity and trustworthiness of the results via this thorough review of equations (5), (6).

(5) $$\Delta Y_{it}=\beta_{i}+\delta_{i}y_{i,t-1}+\lambda_{i}{\bar{y}}_{t-1}+\sum_{j=0}^{p}\psi \Delta {\bar{y}}_{t-j}+\sum_{j=1}^{p}\theta_{ij}\Delta y_{i,t-j}+\mu_{it}$$

(6) $$G_{t}=\frac{1}{N}\sum_{i=1}^{N}\;\frac{\delta_{i}}{SE\left({\hat{\delta }}_{i}\right)}\&G_{a}=\frac{1}{N}\sum_{i=1}^{N}\;\frac{T\delta_{i}}{1-\sum_{j=1}^{k}\;{\hat{\delta }}_{ij}}$$

A dynamic panel model that depicts the connection between a collection of explanatory factors and the change in the dependent variable $\Delta Y_{it}$ It is represented by equation (5). Unobserved influences influencing the change in the dependent variable are captured by the error term. $\mu_{it}$. Two indices, $G_{t}$ and $G_{a}$, are introduced in equation (6) and are computed across the N individual units on the panel. $G_{t}$ gives information on the average impact of the time-specific effects and is calculated by dividing the average value of $\delta_{i}$ by the standard error of $\hat{\delta }$ ^I^ [36]. Conversely, $G_{a}$ provides an estimate of the average influence of individual-specific effects and is defined as T times $\delta_{i}$ divided by (1 minus the total of the delayed coefficients (${\hat{\delta }}_{ij}$ Up to order k). The importance and degree of time- and individual-specific impacts throughout the panel may be determined using these indices. All in all, this framework makes it possible to analyze the dynamic relationships in the panel data in great detail, taking into account both one-time and one-to-one impacts on the dependent variable.

(7) $$P_{t}=\frac{{\hat{\delta }}_{i}}{\text{SE}\left({\hat{\delta }}_{i}\right)}andP_{a}=T\hat{\delta }$$

Two indices, $P_{t}$ and $P_{a}$, are introduced in Equation (7) and are calculated to evaluate the size and relevance of the individual-specific and time-specific effects in the panel data model. The ratio of the individual-specific impact ${\hat{\delta }}_{i}$ to its standard error $\text{SE}\left({\hat{\delta }}_{i}\right)$ yields the index $P_{t}$. If the time-specific effects have a significant influence on the dependent variable, this index sheds light on their statistical significance. Alternatively $P_{a}$ may be calculated [37]. The importance and impact of the individual-specific effects on the panel data are gauged by this index. These indices provide a detailed evaluation of the individual and time-specific contributions to the observed changes in the dependent variable, which is useful information for comprehending the statistical significance of the various effects in the dynamic panel model.

(8) $$S_{it}=\sum_{j=1}^{q}\gamma_{\text{ij}}S_{i,t-j}+\sum_{j=1}^{q}\beta_{\text{ij}}^{\prime }x_{i,t-j}+\mu_{i}+\varepsilon_{it}$$

The structural equation for a dynamic panel model, represented by $S_{it}$, is given in equation (8). The temporal dynamics present in the data are captured by this equation, which states that the dependent variable $S_{it}$ is affected by lagged values of itself $(S_{i,t-j}$) up to a particular order q. The influence of lagged values of exogenous variables, which are represented by the vector ($x_{i,t-j})$ is also included in the model. The impacts of these variables on the dependent variable are captured by the coefficients. ${\;}_{\text{ij}}^{\prime }$'. For the ith unit in the panel, the error term $\varepsilon_{it}$ accounts for unobservable causes causing the variance in the dependent variable, whereas $\mu_{i}$ Indicates effects that are particular to a person and do not change over time [38]. Concerning describing the change in the dependent variable over time, this equation offers a framework for examining the structural connections within the panel data, considering both endogenous and exogenous influences in equation (9).

(9) $$\delta S_{it}=\delta_{i}\left(\delta S_{i,t-1}\eta_{i}^{\prime }x_{i,t}\right)\sum_{j=1}^{q-1}\gamma_{\text{it}}\delta y_{i,t-j}+\sum_{j=0}^{q-1}\beta_{\text{ij}}^{\prime }x_{i,t-j}+\mu_{i}+\varepsilon_{it}$$

It is crucial to determine the statistical significance of variable I, which represents the adjustment rate between 0 and 2. The method used in this research may easily accommodate a mixed integration order without facing difficulties, even though it is ideal for the variables to have the same integration order. This opposes the traditional approaches found in the body of current literature. What sets this estimator apart from conventional techniques often used in comparable investigations is its capacity to provide insightful information that encompasses both present and future data [39]. This approach's flexibility allows for a more thorough comprehension of the adjustment rate and integration orders, providing a more nuanced view of the connections between the variables in the research framework.

**Fig. 4:**
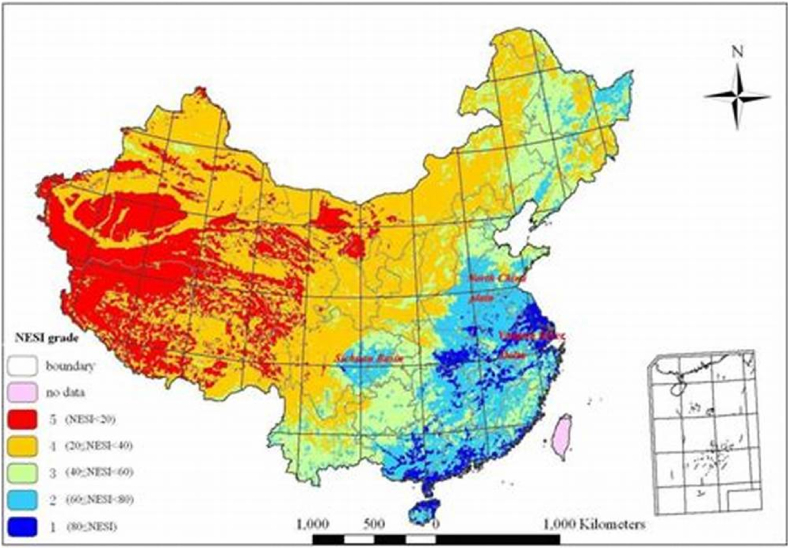
Natural environment sustainability.

The east and south are where China's vegetation thrives, thanks to excellent rainfall amount and warmth, but drier with cold temperatures in the northwest represent its sparser picture. This NDVI map represents an interaction of climate, soil, and human factors, providing a valuable device for evaluating plant health and environmental changes such as drought or deforestation as shown in Fig. 4.

## 4 Result and discussion

### 4.1 Expressive statistics

This part explores the study's analysis and discussion of its results. The study began with a review of the descriptive statistics, which include means, medians, and ranges, which are shown in (Table 2). Positive mean or median values are guided by the variables GDP, TI, and RE, highlighting their progressive nature and supporting the findings [37]. It's interesting to note that, when considering forest rents in the context of China, the mean and median values for Natural Extraction Revenue (NRR) were found to be negative. Furthermore, significant findings were obtained by examining the variables' lowest and maximum values. This suggests that there were periods of economic boom and recession over the analyzed period, as well as variations in the rents associated with natural resources and the advancement of technical innovation in tandem with different levels of urbanization. Descriptive statistics function as a fundamental examination of the data, providing an understanding of the main trends and fluctuations of the variables that are being studied.

**Table 2:** Descriptive statistics.

Var.	N	Unkind	(S.D)	Minimum	Middle	Maximum
log REO	0.088	0.09	−0.066	0.077	0.88	927
Log GNI	1.821	0.651	0.29	1.89	3.09	927
Log RER	19.18	1.46	18.29	18.759	31.051	927
Log TAR	21.771	4.077	3.002	17	41	927
Log UPD	0.05	0.051	−0.071	0.88	0.49	931

Various expressive assessments for distinct variables are shown in (Table 2). The first variable shows a mean value of 0.088 with a standard deviation of 0.09; it is called log REO (Renewable Energy Output). The numbers representing the lowest, medium (median), and highest are −0.066, 0.077, and 0.88, in that order [40]. This suggests a noticeable range of values in the data distribution for producing renewable energy, mostly focused on the median. The Gross National Income (GNI) variable, which is the second variable, has a mean of 1.821 and a standard deviation of 0.651. With a minimum of 0.29 and a high of 3.09, the data range illustrates the variation in gross national income among the units under observation. The mean and standard deviation of the third variable, Log RER (Environmental Resource Rents), are 19.18 and 1.46, respectively. The numbers that represent the lowest, median, and maximum are 18.29, 18.759, and 31.051, in that order.

**Fig. 5:**
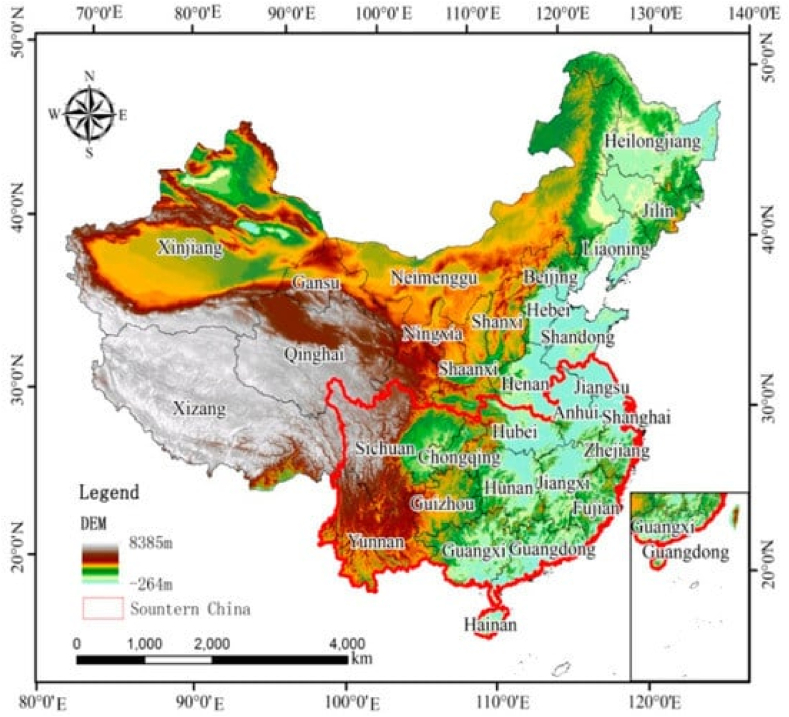
Sustainability.

China's demographic map looks like a colorful downtown disappearing into an enormous countryside. Rich eastern coast with megacities like Shanghai and Beijing thrive with high density, while inland regions show more moderate clusters around economic centers like Chengdu. The expansive west and north, concealed in colors of bareness, show the narrow clutches of hostile surroundings and slim prospects. This stark divide – shaped by economic magnets and geographical constraints – has proven to be more challenging in efforts to narrow regional disparities, accelerate inland growth levels, and conserve fragile ecosystems while dancing these intricate steps of urbanization versus environmental sustainability as shown in Fig. 5.

This points to a distribution that is right-skewed and has a concentration of values around the lower end of the range. Technology Adoption Rates (Log TAR), the fourth variable, has a mean of 21.771 and a standard deviation of 4.077. The observed entities exhibit various technology adoption rates, as shown by the data range of 3.002–41. Finally, the mean and standard deviation of Log UPD (Urban Population Density) are 0.05 and 0.051, respectively. The numbers are −0.071, 0.88, and 0.49 for the lowest, median, and maximum, respectively. The variable has a moderate spread and a relatively small mean, indicating variances in the urban population density among the units under analysis [41]. All in all, these metrics provide a thorough summary of the ranges, variabilities, and central trends for every variable in the dataset.

The examination of dependencies between sections for different variables is shown in (Table 3). With a test statistic of 31.107 and a p-value of 0.003, demonstrated by '***,' the first variable, Log REO (Renewable Energy Output), offers a considerable dependence across sections, showing a high degree of statistical significance. Similarly, with a test statistic of 31.514 and a p-value of 0.004, Log GNI (Gross National Income) demonstrates a significant reliance across sections, highlighting the statistical importance of the observed dependency (H. [42]). The study for Log RER (Environmental Resource Rents) shows cross-sectional dependence, which is further significant, as shown by the test statistic 19.571 and p-value of 0.005. With a test statistic 14.790 and a p-value of 0.008, the variable Log TAR (Technology Adoption Rates) also shows a substantial reliance across sections.

**Table 3:** Cross section dependency.

Var.	Dependency across sections	P(value)
Log REO	31.107***	0.003
Log GNI	31.514***	0.004
Log RER	19.571***	0.005
Log TAR	14.790***	0.008
Log UPD	27.430***	0.12

The Urban Population Density, or Log UPD, exhibits a significant inter-sectional dependence, as shown by a test statistic 27.430 and a p-value of 0.12. The strong statistical significance of the observed dependencies across sections for each variable is highlighted by the constant presence of '***' and the corresponding p-values of 0.003 across all variables (Table 3). Understanding the possible effects of cross-sectional dependencies on the research is largely dependent on these findings, which also highlight the need for suitable statistical modifications in further studies. This part explores the study's analysis and discussion of its results. The study began with a review of the descriptive statistics, which include means, medians, and ranges, which are shown in (Table 3). Positive mean or median values are indicated by the variables GDP, TI, and RE, highlighting their progressive nature and supporting the findings of Irfan et al. (2019). It's interesting to note that, when considering forest rents in the context of China, the mean and median values for Natural Extraction Revenue (NRR) were found to be negative.

Furthermore, significant findings were obtained by examining the variables' lowest and maximum values. This suggests that there were periods of economic boom and recession over the analyzed period, as well as variations in the rents associated with natural resources and the advancement of technical innovation in tandem with different levels of urbanization. Descriptive statistics function as a fundamental examination of the data, providing an understanding of the main trends and fluctuations of the variables that are being studied.

The unit root test results for a range of variables under varying requirements are shown in (Table 4). Test statistics for unit root tests are shown in the first column, "Equal Interrupt," interruption-free. There are interruptions in the second column, "Interrupt and Tendency," but no deterministic pattern. "First-difference Interrupt and Tendency," the fourth column, presents the first differences with interruptions and deterministic trends, whereas the third column, "First-difference Interrupt," examines the first differences with interruptions [43]. The test statistic for the equal interrupt specification in Log REO (Renewable Energy Output) is −4.239. For the first-difference interrupt specification, it gets more negative, providing stronger evidence against the existence of a unit root. The statistical significance of the p-values, shown by '***,' is strong since they are less than 0.01. The test's direction, "A (3)," suggests that the alternative hypothesis, that is, that the series has a unit root after the third differencing, is accepted. Log RER (environmental resource rents), Log TAD (technology adoption rates), Log UPD (urban population density), and Log GNI (gross national income) all show similar trends. When initial differences are taken into account, the test statistics in every instance grow more negative, hence confirming the rejection of the unit root null hypothesis. The strong statistical significance of the data is shown by the persistent appearance of '***' in the p-values. Overall, the unit root tests indicate that, upon suitable differencing, the variables under investigation show stationarity features, offering crucial information for time-series research.

**Table 4:** Unit root test.

Var.	Equal		First-difference		Direction
	Interrupt	Interrupt and tendency	Interrupt	Interrupt and tendency	
(CIPS)					
Log REO	−4.239	−4.669	−5.631***	−5.690***	A(3)
Log GNI	−4.888	−4.581	−3.959***	−4.105***	A(3)
Log RER	−1.447	−3.203	−3.104***	−3.959***	A(3)
Log TAD	−3.649	−3.888	−5.831***	−7.950***	A(3)
Log UPD	−3.361	−3.888	−2.719***	−3.803***	A(3)

The cointegration analysis results are shown in (Table 5), which offers a comprehensive analysis of the statistical significance of the various test statistics (Ga, Gb, Pa, and Pb) for each specification. Asterisks indicate the significance levels: * denotes statistical significance, ** suggests moderate importance, and *** denotes high relevance. The findings demonstrate how important cointegration tests are for evaluating the long-term connections between variables. Ga and Gb have continuous relevance across several specifications, highlighting the cointegration connections' resilience Hilson & Hu, 2022 [44] Pa and Pb also show different degrees of significance, which adds to the detailed knowledge of the underlying dynamics. These results provide important information on the existence and degree of cointegration linkages and provide a lasting understanding of the links between the variables under study. A thorough assessment of cointegration within the dataset is made possible by the variety of specifications considered in the research.

**Table 5:** Analysis of cointegration.

Test (Statistics)	Significance	Significance	Significance	Significance	Significance	Significance
G_a_	−16.128*	−17.966*	−16.180*	−17.866*	−16.118*	−17.480*
G_b_	−15.966***	−17.247***	−17.111***	−17.248***	−17.839***	−17.871***
P_a_	−17.390***	−17.190***	−15.888***	−17.800**	−14.866***	−18.390***
P_b_	−6.170***	−7.131***	−6.971***	−7.888***	−7.670***	−7.570***

(Table 6) shows the findings of the cross-sectional autoregressive distributed lag (CS-ARDL) model's short- and long-term analysis. The standard errors for each coefficient estimate are demonstrated by the numbers in parentheses. The coefficients for log REO, log GNI, log RER, log TI, and log UPD are given for the short-term results. Every coefficient has a corresponding standard error. Notably, the symbols '***,' '**,' and '*' signify statistical relevance, moderate importance, and high statistical significance, respectively. The coefficients and standard errors for the same variables are shown in the section on long-term outcomes Ozcan et al., 2023 [45] The findings provide light on how the variables relate to one another across both short- and long-term timescales, which is important knowledge for comprehending the system's dynamics under study. The analysis is more reliable and resilient when the significance levels corresponding to the coefficient estimations are considered.

**Table 6:** Short and long-run analysis of (CS-ARDL).

	Values	Values
Short-term outcomes		
log REO	−0.148*** (0.051)	−0.171*** (0.049)
Log GNI	0.488*** (0.161)	0.477*** (0.159)
Log RER	−0.061** (0.041)	−0.029** (0.017)
logTI	0.980*** (0.166)	0.819*** (0.190)
Log UPD	−0.166 (0.141)	
Long-term outcomes		
Log REO	−0.180*** (0.071)	−0.190*** (0.039)
Log GNI	0.588*** (0.241)	0.677*** (0.255)
Log RER	−0.929*** (0.088)	−0.866*** (0.071)
Log TAR	0.988*** (0.318)	0.766*** (0.290)
Log UPD	−0.088*** (0.050)	

### 4.2 Robust analysis

The findings of a robustness study using trade and economic globalization indices are shown in (Table 7) in the context of both short- and long-term outcomes. The standard errors for each coefficient estimate are demonstrated by the numbers in parentheses. The coefficients for log REO, log GNI, log RER, log TAR, and log UPD are given for the short-term results, along with the corresponding standard errors. The asterisks ('***' for high significance, '**' for moderate significance, and '*' for statistical relevance) show the degree of statistical significance. Similar coefficients and standard errors for log REO, log GNI, log RER, log TAR, and log UPD are provided in the section on long-term outcomes Limi Kouotou & Atangana Ondoa, 2023 [46] Considering the impact of trade and economic globalization indices, the findings provide insight into the links between the variables from both short- and long-term perspectives. The coefficients' significance levels enhance the analysis's resilience and dependability while offering a thorough grasp of the system's dynamics under study across various analytical paradigms.

**Table 7:** Utilizing trade and economic globalization indexes for robustness analysis.

Var.	Values	Values	Values	Values
Short-term outcomes				
log REO	−0.181 (0.224)		−0.171 (0.119)	
Log GNI	−0.039*** (0.014)			−0.050*** (0.007)
Log RER		−0.141*** (0.051)	−0.161*** (0.059)	
Log TAR	0.566*** (0.161)	0.690*** (0.229)	0.739*** (0.259)	0.550*** (0.171)
Log UPD	0.959*** (0.239)	0.841*** (0.213)	0.990*** (0.251)	0.890*** (0.231)
Long–run outcomes				
log REO	−0.088*** (0.049)		−0.117** (0.049)	
Log GNI	−0.051** (0.019)			−0.051** (0.018)
Log RER		−0.259*** (0.048)	−0.288*** (0.066)	
Log TAR	0.877*** (0.350)	0.588*** (0.177)	0.679*** (0.290)	0.821*** (0.277)
Log UPD	3.104*** (0.249)	0.821*** (0.231)	3.124*** (0.339)	0.829*** (0.161)

The results of a robustness study using the AMG (Amplified Mean Group) analysis are shown in (Table 8). The coefficients for the various variables are represented by the numbers in the table, and each coefficient estimate's degree of statistical significance is indicated by the asterisks. The coefficients for log REO are shown for several specifications, demonstrating how sensitive the outcomes are to changes in the analytical methodology [47]. The statistical significance levels are indicated by the asterisks, which include '***' and '**,' where '***' denotes high significance and '**' indicates moderate significance. Coefficients for log GNI, log RER, log TAD, and log UPD are also given, emphasizing how sensitive they are to modifications in the analytical process. The robustness of the outcomes from the AMG analysis is partly evaluated by the significance levels connected to the coefficients. With the AMG technique, this robustness analysis provides a thorough knowledge of the stability and dependability of the analytical findings by shedding light on how methodological differences may affect the calculated coefficients.

**Table 8:** Analyzing robustness using the AMG analysis.

Var.	values	Values	Values	Values	Values	Values
log REO	−0.049***		−0.071**		−0.080***	
Log GNI	−0.191***					−0.261**
Log RER		−0.041**	−0.041**			
Log TAD				−0.261***	−0.266***	
Log UPD	0.970***	0.790***	0.990***	0.688***	0.721***	0.690***

### 4.3 Heterogeneity

Significant geological differences characterize China's large and diversified environment, resulting in an unequal distribution of mineral richness across areas. Each region, from the deserts of Xinjiang to the plateaus of Inner Mongolia, presents unique mineral kinds and abundances, adding to the complexity of the country's resource environment. Moreover, the governance system of China introduces an additional layer of variability by combining decentralized and centralized decision-making. How mineral wealth is mined and maintained is influenced by the diverse responsibilities that different governmental levels play in resource management.

Because various minerals need other extraction processes and processing methods may have distinct ecological effects, the environmental effects of mineral extraction are also heterogeneous. In a larger sense, various elements, such as cultural norms, economic policies, and governance practices, influence how the "curse of the natural resource" manifests itself. China's encounter with the curse of natural resources will probably vary depending on the location, kind of mineral, and level of resource extraction ([[29], [48], [49], [50]]). The title effectively summarizes the complex interactions between geology, governance, environmental, and contextual issues, highlighting the necessity for a detailed analysis of China's complicated relationship with its mineral richness and the difficulties presented by the curse of natural resources.

The statistical results for the slope of heterogeneity for the two variables, Δ∼ and Familiar, are shown in (Table 9). Asterisks indicate statistical significance; three asterisks (***) signify a high degree of relevance. The range of values for Familiar is 15.788–17.488, and for Δ∼ is 17.314–21.206. Three asterisks are consistently present for every value, which suggests a strong statistical significance. These slope values are important measures of the dataset's degree of heterogeneity. The variable Δ∼ has somewhat higher values than Familiar, indicating a possibly higher variance related to Δ∼. The statistical significance highlights the relevance of considering the variation in these slope values and supports the validity of these results. These findings may help analysts and researchers get a deeper comprehension of the variability in the dataset and provide more nuanced insights on the variables portrayed by Δ∼ and Familiar and how they affect the analysis or study as a whole.

**Table 9:** Slope of heterogeneity.

Stat.			Values			
(Δ∼)	17.314***	16.881***	17.610***	18.459***	17.255***	21.206***
(Familiar)	16.361***	15.788***	16.266***	16.366***	17.139***	17.488***

## 5 Conclusion and policy recommendations

This study sheds light on the environmental consequences of China's mineral wealth and governance from 1990 to 2022, utilizing an econometric model to explore these dynamics comprehensively. The analysis reveals that regions with greater mineral wealth have experienced significant environmental degradation, particularly in air and water quality, underscoring the detrimental impact of natural resource abundance when not managed sustainably. The study also highlights the pivotal role of governance, demonstrating that regions with stronger governance frameworks experience less environmental harm, suggesting that good governance can mitigate some of the negative effects associated with mineral wealth. Despite economic growth driven by mineral wealth, there is a notable disconnect between economic and environmental outcomes, indicating that economic gains do not automatically lead to improved environmental conditions.

Policy recommendations emerging from this study emphasize the need for China to strengthen its governance mechanisms and integrate environmental considerations into its resource management strategies. Specifically, policies should focus on improving transparency, accountability, and enforcement of environmental regulations to ensure that economic development does not come at the expense of environmental sustainability. Additionally, investments in cleaner technologies and practices in the mining sector are crucial to reducing the environmental footprint of mineral extraction and processing activities.

The study's limitations include the focus on China, which may limit the generalizability of the findings to other countries with different institutional and economic contexts. Furthermore, the econometric model, while robust, may not capture all the complex interactions between mineral wealth, governance, and environmental outcomes. Future research should explore similar dynamics in other resource-rich countries to validate and extend the findings of this study. Additionally, further investigation into the specific mechanisms through which governance influences environmental outcomes would provide deeper insights into effective policy interventions. Expanding the temporal scope to include more recent data and incorporating other environmental indicators could also enhance the understanding of the long-term environmental impacts of mineral wealth and governance.

## CRediT authorship contribution statement

**Heshu Qiu:** Formal analysis, Data curation, Conceptualization, Zhen Liu, Conceptualization.

## Declaration of competing interest

The authors declare that they have no known competing financial interests or personal relationships that could have appeared to influence the work reported in this paper.

## References

[1] Ullah M., Umair M., Sohag K., Mariev O., Khan M.A., Sohail H.M. (2024). The connection between disaggregate energy use and export sophistication: new insights from OECD with robust panel estimations. Energy.

[2] Chen J.M., Umair M., Hu J. (2024). Green finance and renewable energy growth in developing nations: a GMM analysis. Heliyon.

[3] Hussain A., Umair M., Khan S., Alonazi W.B., Almutairi S.S., Malik A. (2024). Exploring sustainable healthcare: innovations in health economics, social policy, and management. Heliyon.

[4] Yiming W., Xun L., Umair M., Aizhan A. (2024). COVID-19 and the transformation of emerging economies: financialization, green bonds, and stock market volatility. Resour. Pol..

[5] Shi H., Umair M. (2024). Balancing agricultural production and environmental sustainability: based on economic analysis from north China plain. Environ. Res..

[6] Xinxin C., Umair M., Rahman S. ur, Alraey Y. (2024). The potential impact of digital economy on energy poverty in the context of Chinese provinces. Heliyon.

[7] Dilanchiev A., Umair M., Haroon M. (2024). How causality impacts the renewable energy, carbon emissions, and economic growth nexus in the South Caucasus Countries?. Environ. Sci. Pollut. Control Ser..

[8] Yu M., Wang Y., Umair M. (2024). Minor mining, major influence: economic implications and policy challenges of artisanal gold mining. Resour. Pol..

[9] Li H., Chen C., Umair M. (2023). Green finance, enterprise energy efficiency, and green total factor productivity: evidence from China. Sustainability.

[10] Zhang W., Liu X., Wang D., Zhou J. (2022). Digital economy and carbon emission performance: evidence at China's city level. Energy Pol..

[11] Mohsin Muhammad, Dilanchiev Azer U.M. (2023). The impact of green climate fund portfolio structure on green finance: empirical evidence from EU countries. Ekonomika.

[12] Yuan H., Zhao L., Umair M. (2023). Crude oil security in a turbulent world: China's geopolitical dilemmas and opportunities. Extr. Ind. Soc..

[13] Wu Q., Yan D., Umair M. (2023). Assessing the role of competitive intelligence and practices of dynamic capabilities in business accommodation of SMEs. Econ. Anal. Pol..

[14] Yan J., Haroon M. (2023). Financing efficiency in natural resource markets mobilizing private and public capital for a green recovery. Resour. Pol..

[15] Yu M., Umair M., Oskenbayev Y., Karabayeva Z. (2023). Exploring the nexus between monetary uncertainty and volatility in global crude oil: a contemporary approach of regime-switching. Resour. Pol..

[16] Ahmad M., Jiang P., Majeed A., Umar M., Khan Z., Muhammad S. (2020). The dynamic impact of natural resources, technological innovations and economic growth on ecological footprint: an advanced panel data estimation. Resour. Pol..

[17] Cui X., Umair M., Ibragimove Gayratovich G., Dilanchiev A. (2023). DO remittances mitigate poverty? AN empirical evidence from 15 selected asian economies. Singapore Econ. Rev..

[18] Li C., Umair M. (2023). Does green finance development goals affects renewable energy in China. Renew. Energy.

[19] Liu F., Umair M., Gao J. (2023). Assessing oil price volatility co-movement with stock market volatility through quantile regression approach. Resour. Pol..

[20] Umair M., Dilanchiev A. (2022).

[21] Umar M., Ji X., Kirikkaleli D., Shahbaz M., Zhou X. (2020). Environmental cost of natural resources utilization and economic growth: can China shift some burden through globalization for sustainable development?. Sustain. Dev..

[22] Zhang Y., Umair M. (2023). Examining the interconnectedness of green finance: an analysis of dynamic spillover effects among green bonds, renewable energy, and carbon markets. Environ. Sci. Pollut. Control Ser..

[23] Li M., Hamawandy N.M., Wahid F., Rjoub H., Bao Z. (2021). Renewable energy resources investment and green finance: evidence from China. Resour. Pol..

[24] Xiuzhen X., Zheng W., Umair M. (2022). Testing the fluctuations of oil resource price volatility: a hurdle for economic recovery. Resour. Pol..

[25] Liang Y., Galiano J.C., Zhou H. (2023). The environmental impact of stock market capitalization and energy transition: natural resource dynamics and international trade. Util. Pol..

[26] Manaugh K., El-Geneidy A. (2011). Validating walkability indices: how do different households respond to the walkability of their neighborhood?. Transp. Res. D Transp. Environ..

[27] Liu H., Alharthi M., Atil A., Zafar M.W., Khan I. (2022). A non-linear analysis of the impacts of natural resources and education on environmental quality: green energy and its role in the future. Resour. Pol..

[28] Asif M., Khan K.B., Anser M.K., Nassani A.A., Abro M.M.Q., Zaman K. (2020). Dynamic interaction between financial development and natural resources: evaluating the ‘Resource curse’ hypothesis. Resour. Pol..

[29] Yu Y. (2023). Role of Natural resources rent on economic growth: fresh empirical insight from selected developing economies. Resour. Pol..

[30] Li Y., Mehmood N., Iqbal N. (2022). Natural resource abundance and financial development: a case study of emerging (E−15) economies. Resour. Pol..

[31] Kongbuamai N., Bui Q., Yousaf H.M.A.U., Liu Y. (2020). The impact of tourism and natural resources on the ecological footprint: a case study of ASEAN countries. Environ. Sci. Pollut. Control Ser..

[32] Lei X., Alharthi M., Ahmad I., Aziz B., Abdin Z. ul (2022). Importance of international relations for the promotion of renewable energy, preservation of natural resources and environment: empirics from SEA nations. Renew. Energy.

[33] Shen Y., Su Z.W., Malik M.Y., Umar M., Khan Z., Khan M. (2021). Does green investment, financial development and natural resources rent limit carbon emissions? A provincial panel analysis of China. Sci. Total Environ..

[34] Julio N., Figueroa R., Ponce Oliva R.D. (2021). Water resources and governance approaches: insights for achieving water security. Water (Switzerland).

[35] Omri A., Bel Hadj T. (2020). Foreign investment and air pollution: do good governance and technological innovation matter?. Environ. Res..

[36] Kim W.S., Kiymaz H., Oh S. (2020). Do country-level legal, corporate governance, and cultural characteristics influence the relationship between insider ownership and dividend policy?. Pac. Basin Finance J..

[37] Van Assche K., Gruezmacher M., Vodden K., Gibson R., Deacon L. (2021). Reinvention paths and reinvention paradox: strategic change in Western Newfoundland communities. Futures.

[38] Yang Y., Khan A. (2021). Exploring the role of finance, natural resources, and governance on the environment and economic growth in South Asian countries. Environ. Sci. Pollut. Control Ser..

[39] Le T.H., Park D. (2021). What drives energy insecurity across the world? A panel data analysis. Energy Res. Social Sci..

[40] Khaled R., Ali H., Mohamed E.K.A. (2021). The Sustainable Development Goals and corporate sustainability performance: mapping, extent and determinants. J. Clean. Prod..

[41] Pedersen L.H., Fitzgibbons S., Pomorski L. (2021). Responsible investing: the ESG-efficient frontier. J. Financ. Econ..

[42] Wu H., Hao Y., Ren S., Yang X., Xie G. (2021). Does internet development improve green total factor energy efficiency? Evidence from China. Energy Pol..

[43] Mohan P., Strobl E., Watson P. (2021). Innovation, market failures and policy implications of KIBS firms: the case of Trinidad and Tobago's oil and gas sector. Energy Pol..

[44] Hilson G., Hu Y. (2022). Changing priorities, shifting narratives: remapping rural livelihoods in Africa's artisanal and small-scale mining sector. J. Rural Stud..

[45] Ozcan B., Temiz M., Gültekin Tarla E. (2023). The resource curse phenomenon in the case of precious metals: a panel evidence from top 19 exporting countries. Resour. Pol..

[46] Limi Kouotou H., Atangana Ondoa H. (2023). Africa's natural resource curse: does the arrival of new heads of state break the spell?. Resour. Pol..

[47] Khan Z., Hossain M.R., Badeeb R.A., Zhang C. (2023). Aggregate and disaggregate impact of natural resources on economic performance: role of green growth and human capital. Resour. Pol..

[48] Li Y., Umair M. (2023). The protective nature of gold during times of oil price volatility: an analysis of the COVID-19 pandemic. Extr. Ind. Soc..

[49] Liu H., Saleem M.M., Al-Faryan M.A.S., Khan I., Zafar M.W. (2022). Impact of governance and globalization on natural resources volatility: the role of financial development in the Middle East North Africa countries. Resour. Pol..

[50] Van Assche K., Verschraegen G., Gruezmacher M. (2021). Strategy for collectives and common goods: coordinating strategy, long-term perspectives and policy domains in governance. Futures.

